# Anticipating Spring: Wild Populations of Great Tits (*Parus major*) Differ in Expression of Key Genes for Photoperiodic Time Measurement

**DOI:** 10.1371/journal.pone.0034997

**Published:** 2012-04-23

**Authors:** Nicole Perfito, Sun Young Jeong, Bengt Silverin, Rebecca M. Calisi, George E. Bentley, Michaela Hau

**Affiliations:** 1 Department of Integrative Biology, University of California, Berkeley, California, United States of America; 2 Department of Migration and Immuno-ecology, Max Planck Institute for Ornithology, Radolfzell, Germany; 3 Department of Zoology, Göthenburg University, Göteborg, Sweden; 4 Helen Wills Neuroscience Institute, University of California, Berkeley, California, United States of America; Institut National de la Recherche Agronomique-CNRS UMR6175, France

## Abstract

Measuring day length is critical for timing annual changes in physiology and behavior in many species. Recently, rapid changes in several photoperiodically-controlled genes following exposure to a single long day have been described. Components of this ‘first day release’ model have so far only been tested in highly domesticated species: quail, sheep, goats and rodents. Because artificial selection accompanying domestication acts on genes related to photoperiodicity, we must also study this phenomenon in wild organisms for it to be accepted as universal. In a songbird, the great tit (*Parus major*), we tested whether a) these genes are involved in photoperiodic time measurement (PTM) in a wild species, and b) whether predictable species and population differences in expression patterns exist. Using quantitative RT-PCR, we compared gene expression after a single long day in male great tits from Sweden (57°42′N) with that from a German (47°43′N) population. Hypothalamic gene expression key for PTM changed only in the northern population, and occurred earlier after dawn during the single long day than demonstrated in quail; however, gonadotropins (secretion and synthesis) were stimulated in both populations, albeit with different timing. Our data are the first to show acute changes in gene expression in response to photostimulation in any wild species not selected for study of photoperiodism. The pronounced differences in gene expression in response to a single long day between two populations raise exciting new questions about potential environmental selection on photoperiodic cue sensitivity.

## Introduction

Seasonally breeding animals have evolved accurate time-keeping mechanisms to pattern transitions through their annual life cycle. Periods of high energetic demand are timed to coincide with favorable environmental conditions (e.g., to produce young when food is abundant). For mid- to high- latitudes, abundant food resources typically occur in spring when ambient temperatures increase and plant and animal productivity increases by orders of magnitude. Accordingly, for many birds and small mammals with short gestation/incubation times, breeding begins in late winter and early spring during long days (‘long-day breeders’), while in mammals with gestation periods closer to six months, breeding occurs during short days in fall and winter (‘short-day breeders’). Measurement of day length by photoperiodic organisms is critical for timing physiological transitions between life-history stages. Day length is a highly predictable cue that allows individuals to anticipate changing conditions well in advance [1]–[6]. An enormous amount of research has been devoted to understanding the molecular basis of seasonal time measurement, but only recently has a model been proposed that incorporates rapid (within hours) changes in gene expression [7], [8]. This model was first developed for a domesticated bird species, Japanese quail, *Coturnix japonica* [9], extended in photoperiodic rodents [10], [11], and later proposed to act over a longer time scale (days) and in the opposite direction in domesticated sheep [12], [13] and in goats [14]. As a result, the changes in gene expression observed in response to a long day stimulus have been proposed to be components of an ancestral mechanism for photoperiodic time measurement (PTM).

Here, we provide the first test of this model in a wild avian species, the European great tit (*Parus major*). Unlike photoperiodic mammals, birds are thought to detect day length using photoreceptive cells within the brain [15]–[17]. Cells containing the photopigments rhodopsin [18]–[20], neuropsin (Opn5) [21], [22], melanopsin (Opn4) [23] and vertebrate ancient opsin (VA opsin) [24], [25] have all been implicated as putative photosensitive cells, and recent studies suggest VA opsin [24], [25] and Opn5 [21] may play important roles in PTM. It is still unclear exactly how information from photoreceptive cells is communicated to other parts of the brain, but stimulatory long photoperiods produce a complex physiological cascade that eventually leads to gonadotropin releasing hormone (GnRH) secretion from nerve terminals at the median eminence within the medial basal hypothalamus (MBH). Using microarrays, Nakao and colleagues [7] determined the temporal patterns of gene expression in response to one long day in the MBH as well as the pars tuberalis (PT) of the pituitary gland in quail. Together with this and other studies, Yoshimura and Sharp [8] have proposed the following ‘first day release’ model for photoperiodic activation of the reproductive system, although these researchers did not investigate changes in GnRH expression.

In quail, circulating LH increases 20 hours after dawn of the first long day [26], [27]. The earliest detectable change in gene expression after a single long day is an increase in the beta subunit of thyroid stimulating hormone (TSHβ) in the PT 14 hours after dawn. The resulting TSH is thought to bind to its receptors expressed in the hypothalamus 4 hours later causing up-regulation of type II iodothyronine deiodinase (DIO2) and down-regulation of type III iodothyronine deiodinase (DIO3). DIO2 and DIO3 are thyroid hormone metabolizing enzymes; DIO2 converts the prohormone thyroxine (T4) to its bioactive form, triiodothyronine (T3), and DIO3 inactivates T3 via conversion to T2 or by converting T4 to reverse T3 [13]. Reciprocal switching of DIO2 and DIO3 gene activation is thought to cause a local increase in T3 availability in the MBH. Central infusion or implants of T3 in the MBH cause gonadal growth on short days [28], perhaps through retraction of glial endfeet encasement around GnRH nerve terminals allowing access of terminals and their contents to the basal lamina and portal blood supply to the pituitary [29]–[31]. Therefore, the local increase of T3 is thought to play a pivotal role in GnRH release, although it is still not clear if or how this local production of T3 influences GnRH synthesis in GnRH neurons, which lie in the pre-optic area (POA), some distance from the median eminence.

The physiological cascade described above can only be triggered if light cues are present during a specific time window (called the ‘photoinducible phase’) during the light cycle. For quail, the photoinducible phase begins at approximately 12 hours after dawn (the ‘critical photoperiod’) and ends approximately 4 hours later [32], [33]. Clock genes involved in circadian time measurement (e.g. Cry, Per) are thought to give seasonal information, and perhaps are involved in the ability to respond to light when it occurs during the photoinducible phase [34]. The critical photoperiod is population-specific and correlates well with latitude of origin [35], [36].

Components of this model appear to be conserved in both mammals and birds, but to date, mainly domesticated species have been used to test the acute changes in gene expression used for PTM, and the changes in photoperiodic ungulates occur over days rather than hours in quail [7], [12], [37]. Critically, these species have been artificially selected for traits related to photoperiodicity, so it is not clear if this system exists in wild species and, if so, how it acts. A recent study comparing whole genomes of domesticated chicken strains demonstrates a complete loss of diversity in the sequence of the TSH-receptor specifically [38], which likely reflects artificial selection on traits related to photoperiodicity. Thus, if we are to understand if mechanisms such as the DIO2/DIO3 response are evolutionarily conserved, we must study them in wild organisms in addition to domesticated species. One wild species that has been extensively studied in many aspects of its ecology including reproductive timing is the great tit (*Parus major*). It is largely sedentary and broadly distributed throughout Europe. Sensitivity of great tit populations to long day lengths (i.e., critical photoperiod) is known to differ with latitude [36] resulting in population variation in the onset of gonad growth. Northern populations of great tits breed later in the year in the field and require a longer day length to activate the reproductive axis in the laboratory compared to birds from southern Europe.

We took advantage of the well-known reproductive ecology and physiology of great tits to test whether a) the proposed ‘first-day release model’ applies to wild vertebrates and b) gene expression in the MBH and circulating concentrations of luteinizing hormone (LH) over one long day differ in a predictable way between birds captured in Sweden (57°42′N) and those in Germany (47°43′N) and kept under controlled conditions. We predicted that changes in the expression of genes involved in PTM should occur later in the Swedish population, matching their longer critical photoperiod and later breeding times, compared to the southern German population. We brought wild great tit males from both populations captured in the fall into common garden conditions and exposed them to a single long day, collecting tissue and blood samples for gene expression and circulating LH concentrations at intervals throughout that day.

## Results

### Morphology

German and Swedish males were similar in body size (tarsus: 23.21±0.79 mm German and 23.19±1.01 mm Swedish; *t*_(64)_ = 0.12, *p* = 0.90), mass (20.32±1.60 g German and 20.50±1.13 g Swedish; *t*_(64)_ = 0.52, *p* = 0.61), and had similar fat scores (2.15±0.49 German and 2.29±0.57 Swedish; *t*_(64)_ = 1.08, *p* = 0.28) at the end of the experiment. Likewise, testis length (0.51±0.39 mm German and 0.51±0.44mm Swedish; *t*_(67)_ = 0.37, *p* = 0.97) and volume (0.08±0.12 mm^2^ German and 0.14±0.25 mm^2^ Swedish; *t*_(67)_ = 1.23, *p* = 0.22) were similar between populations.

### Clock gene mRNA expression

**Figure 1:**
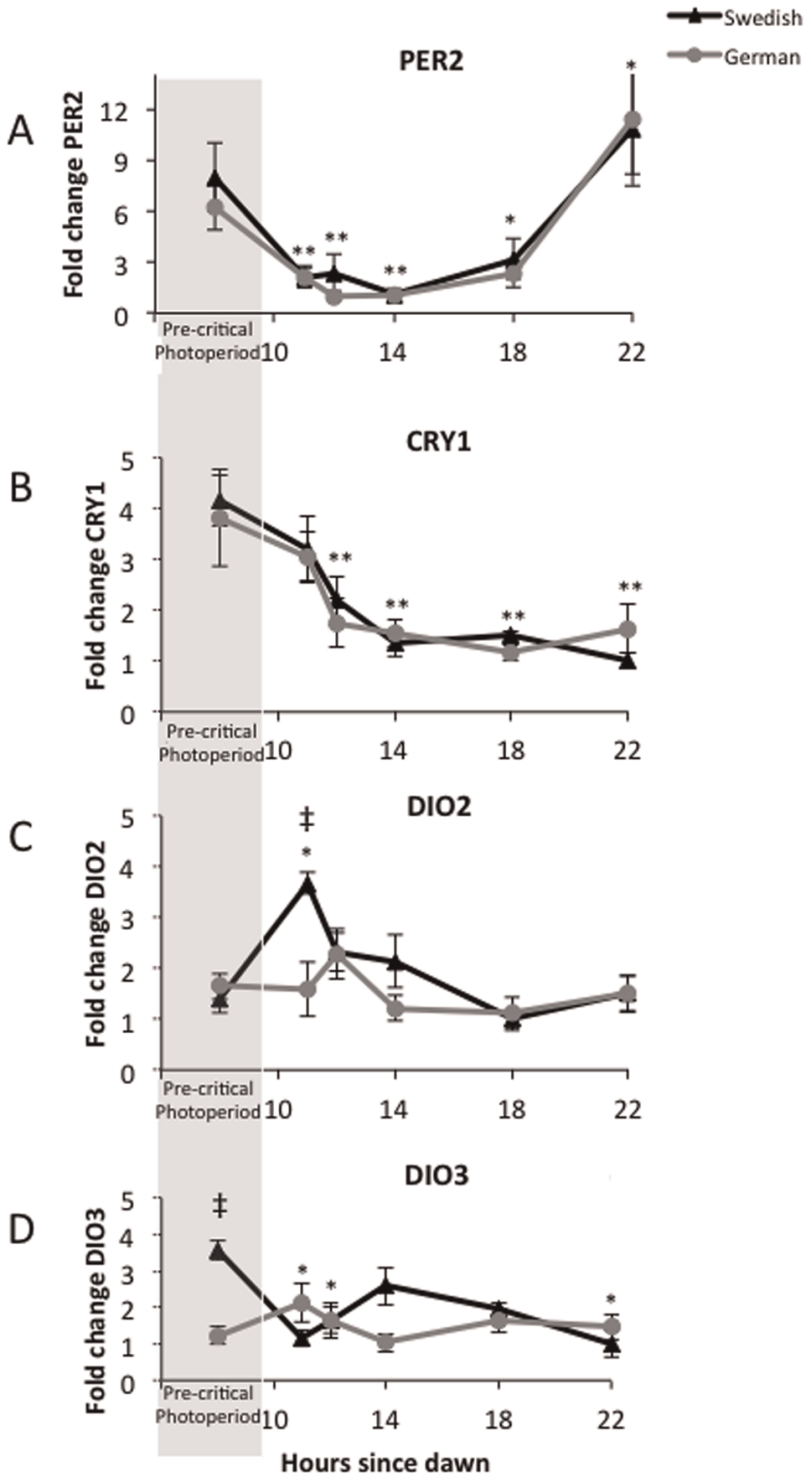
Fold change in mRNA expression of PER2 (**A**), **CRY1** (**B**), **DIO2** (**C**) **and DIO3** (**D**) **relative to control genes during exposure to one long day in German and Swedish photosensitive great tit males.** Both clock genes show similar expression between populations. DIO2 expression increased and DIO3 expression decreased at 11 hours after dawn following pre-threshold sampling times (gray bar) in the Swedish population only. The symbols ** indicate both populations differ from pre-threshold values, and * indicates a single population differs from pre-threshold values, ‡ indicates populations differ significantly from each other.

Populations showed similar mRNA expression patterns over time for both of the clock gene proteins. Per2 expression decreased in both populations during post critical photoperiod time points (p<0.05) until 14 hours after dawn. Although populations had similar expression of Per2 at 18 and 22 hours after dawn, only the Swedish population had significantly lower Per2 expression relative to pre-critical photoperiod levels at 18 hours (p = 0.021) and the German population had slightly higher Per2 expression at 22 hours after dawn (p = 0.02; Fig. 1A). Expression of Cry1 decreased from pre-critical photoperiod levels similarly in both populations (Fig. 1B). Expression levels were significantly different from pre-critical photoperiod levels at 12, 14, 18 and 22 hours after dawn for both German and Swedish birds (p<0.02).

### Thyroid metabolizing enzymes mRNA expression

In the Swedish population, expression of DIO2 mRNA increased from pre-critical photoperiod levels at 11 hours after dawn (t_(56)_ = 5.04, p<0.001), was significantly higher than in the German birds (t_(56)_ = 4.40, p<0.001), and remained somewhat elevated at 12 hours after dawn (p = 0.053; Fig. 1C). In the German population, DIO2 expression did not change significantly from pre-critical photoperiod levels (p>0.05) at any sampling point. Expression of DIO3 mRNA was higher pre-critical photoperiod in the Swedish than the German population (t_(56)_ = 2.88, p = 0.006), and decreased 11 and 22 hours after dawn (p = 0.03 and 0.01) in the Swedish population, indicating reciprocal switching of DIO2 and DIO3 expression at 11 hours after dawn (Fig. 1D). In the German population, DIO3 expression did not change significantly from pre-critical photoperiod levels (p>0.05) at any sampling point.

### GnRH mRNA expression

**Figure 2:**
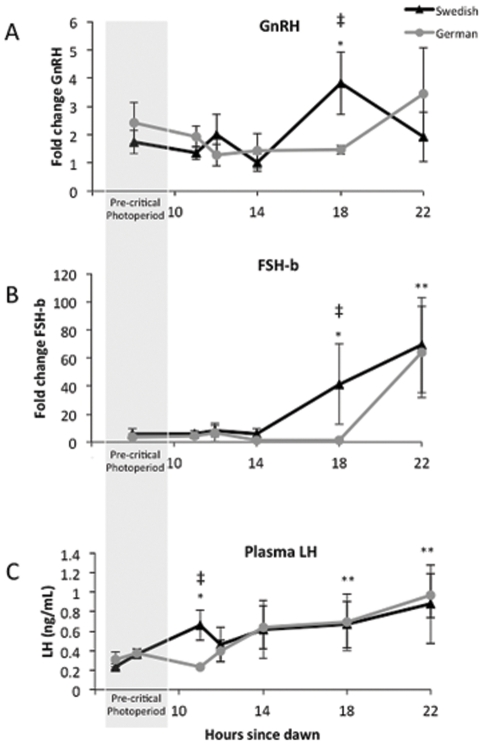
Fold change in mRNA expression of GnRH in the brain (**A**) **and FSH** ***ß*** (**B**) **in the anterior pituitary gland relative to control genes during exposure to one long day in German and Swedish photosensitive great tit males.** GnRH increases expression 18 hours after dawn in the Swedish population only, while FSH*ß* stimulated in the Swedish population at 18 hours and both populations by 22 hours after dawn. Circulating LH concentration (C) during exposure to one long day in German and Swedish photosensitive great tit males. LH concentration increased at 11 hours after dawn in the Swedish population, and slowly increases in both populations by 18 hours after dawn. The symbols ** indicate both populations differ from pre-threshold values, and * indicates a single population differs from pre-threshold values, ‡ indicates populations differ significantly from each other.

In the Swedish population, expression of GnRH mRNA increased 18 hours after dawn from pre-critical photoperiod levels (t_(56)_ = 2.27, p = 0.03) and was significantly greater than the German population (t_(56)_ = 2.38, p = 0.02; Fig. 2A). In the German population, GnRH expression did not change significantly from pre-critical photoperiod levels (p>0.05) at any sampling point.

**Figure 3:**
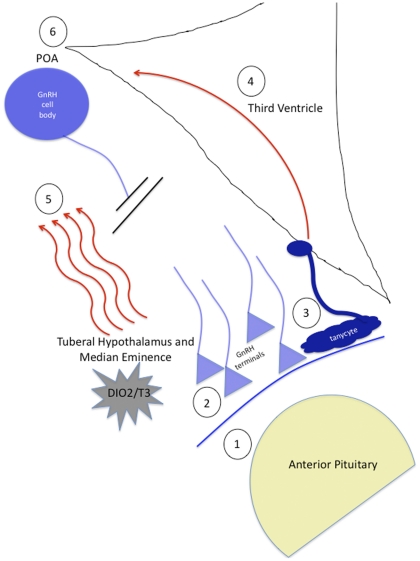
Schematic demonstrating the potential modes of action of T3 to elicit GnRH release at the median eminence (**at 11 hrs**) **and later** (**at 18 hrs**) **on GnRH cell bodies in the preoptic area** (**POA**). T3 could act directly on the pituitary (1) or on GnRH terminals in the ME (2), causing release of GnRH there, or on glial cells causing retraction and then allowing localized release of GnRH in the fiber terminals (3). Several hours later, photostimulation causes up-regulation of GnRH expression potentially from action of T3 on tanycytes surrounding the third ventricle that send a signal to GnRH neurons (4), or from diffusion of locally produced T3 to the POA (5) or via a totally separate mechanism (6).

### Anterior Pituitary FSHβ mRNA expression

In the Swedish population, expression of FSHβ mRNA increased 18 hours after dawn from pre-critical photoperiod levels (t_(61)_ = 5.04, p<0.001; Fig. 2B) and was significantly higher than the German population (t_(61)_ = 5.01, p<0.001 ). By 22 hours after dawn both populations had increased FSHβ mRNA expression from pre-critical photoperiod levels (t_(61)_ = 3.53, p = 0.001 and t_(61)_ = 3.99, p<0.001, German and Swedish populations, respectively).

### Circulating plasma LH

Circulating plasma LH concentration was significantly elevated from pre-bleed concentrations (taken 24 hours before) in the Swedish population (t_(123)_ = 3.64, p<0.001) and was higher than in the German population (t_(123)_ = 2.24, p = 0.03; Fig. 2C) at 11 hours after dawn. Plasma LH increased significantly from pre-bleed levels at 14, 18, and 22 hours after dawn in the Swedish and German population (p<0.04).

## Discussion

Great tits from a Northern European population showed marked changes in gene expression 11 hours after dawn. Expression of DIO2 mRNA increased and DIO3 expression decreased around the presumed critical photoperiod for this species, and the reciprocal switching in expression of these two genes coincided with an increase in LH secretion in the plasma at the same sampling point and a stimulation of GnRH expression six hours later. Our data are the first to show acute changes in gene expression in response to photostimulation in any species not artificially selected for study of photoperiodism. An additional novel component of our experiment is the demonstration of GnRH mRNA activation, which is an important aspect of the first-day release model that has not previously been measured. Further, our data also indicate population differences in the temporal patterns of gene expression under common garden conditions. Interestingly, although reciprocal switching of DIO2/DIO3 occurred only in the Swedish population, *both* populations eventually increased FSHβ expression as well as LH secretion into the plasma. Both processes occurred later after dawn in the German population. Together these data suggest that both populations did respond to photostimulation, but that there are population differences in the speed of response to a long day stimulus. The data from the German population also imply that regulation of DIO2/DIO3 expression is not required for GnRH and/or gonadotropin stimulation, although we state this with the caveat that there is a very small possibility that our sampling schedule caused us to miss a peak in DIO2 or decrease in DIO3 in the German population. Given that we saw population differences at all levels of the pathway (DIO2, DIO3, GnRH, FSHβ, LH), we think this is unlikely. Our study clearly indicates that the DIO2 system is temporally correlated with extremely rapid photoperiodic responses, but that activation of the GnRH neurons, which are located some distance away in the pre-optic area, occurs at a later stage. The combination of the lack of temporal synchronization of DIO2 and GnRH activation along with the physical separation of the DIO2 and GnRH systems implies that photoperiodic up-regulation of GnRH could involve a separate mechanism other than or in addition to the DIO2 system in the mediobasal hypothalamus.

### Gene expression and the first-day release model

The patterns of gene expression in great tit males from the Swedish population are consistent with changes found in quail [7]; however, the timing of expression changes in DIO2 and DIO3 are different relative to the photoperiodic threshold in these two species. In Japanese quail, the photoperiodic threshold occurs around 12 hours after dawn and the photoinducible phase peaks around 15 hours after dawn [39]. Nakao et al. [7] found increased expression of TSHβ in the pars tuberalis within the photoinducible phase (14 hrs after dawn) followed by a sustained reciprocal switching of DIO2 and DIO3 mRNA expression in the MBH 4 hours later (18 hr). Great tits have an earlier photoperiodic threshold, around 10.5–12 hours after dawn [36], [40]. In the great tit, reciprocal switching of DIO2/DIO3 occurs 11 hours after dawn, which is coincident with the threshold for this species and show a more transient change in mRNA expression in contrast to quail. Therefore, the change in expression of thyroid metabolizing enzymes occurred six hours earlier in the wild great tits compared to laboratory bred Japanese quail, and likely reflects different photoinducible phases between species. This is the earliest change in gene expression in response to photostimulation that has been described in any vertebrate. The transient increases in gene expression seen in great tits suggests that this species might require exposure to long days for longer periods of time to activate more persistent changes in DIO2/DIO3 expression.

Our data also indicate that in male great tits the GnRH system is not up-regulated as a result of photostimulation until *after* the initial rise in LH (at 11L). The local action of T3 production likely has a very rapid action in the MBH and may not involve the GnRH neurons in the pre-optic area (POA) during initial photostimulation. The action of DIO2/T3 could be any one, or all of the following: 1) direct action on the stored gonadotropins in the anterior pituitary, 2) on GnRH terminals in the ME, causing release of GnRH there, or 3) on glial cells, causing retraction and then allowing localized release of GnRH in the fiber terminal fields (Fig. 3). Several hours later, photostimulation causes up-regulation of GnRH gene expression and peptide synthesis over a longer time frame. This could potentially occur from: 1) action of T3 on tanycytes surrounding the third ventricle [13] that send a signal to GnRH neurons, or 2) from diffusion of locally produced T3 to the POA or 3) via a totally separate and unknown mechanism altogether (Fig. 3). Given that DIO2 expression continues to be up-regulated in photorefractory (non-breeding) Eurasian tree sparrows when plasma LH concentrations decrease to a minimum [41], and that inhibition of the Dio2 enzyme only slows testis growth, but does not inhibit it completely [42], the latter seems equally plausible. No matter what the mechanism, these findings show us that it is likely that there are important population differences in the way in which great tits integrate changes in day length to their reproductive physiology.

**Figure 4:**
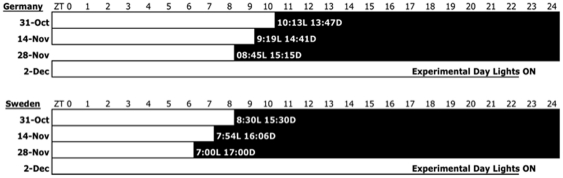
Environmental chamber light schedules for birds captured in Germany (**top panel**) **and from Sweden** (**lower panel**) **at two-week intervals before the experimental day when tissue was collected** (**2-Dec**) **at pre-critical photoperiods** (**6&10 hr after dawn**), **at 11, 12, 14, 18 and 22 hr after dawn.**

**Figure 5:**
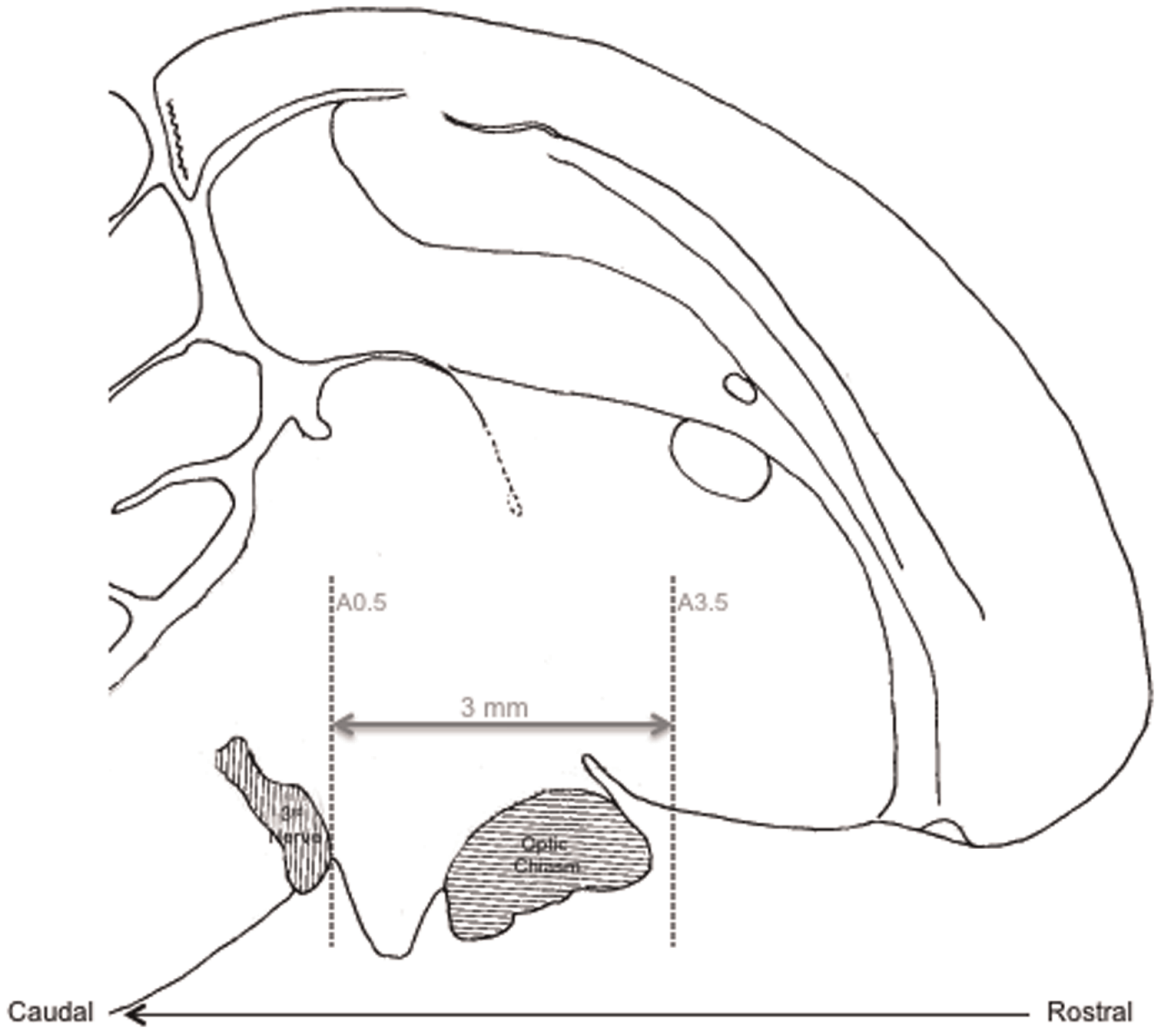
Mid-line sagittal drawing (adapted from [42]) of a passerine brain. Dashed vertical lines represent approximate starting and ending points of tissue punches through the 3 mm of hypothalamus. Tissue punches were collected on alternating right and left sides of each section beginning at approximately A3.5 and ending at approximately A0.5.

### Population differences in gene expression

All of the changes we observed in the proposed hypothalamic photo-transduction pathway occurred in the northern population only. We would have predicted, based on a simple photoperiodic threshold model, that the southern population would show the same pattern of gene expression as the northern population, only slightly earlier in the day. We consider it unlikely that difference in photosensitivity between the two populations at the beginning of our experimental treatment explain these findings. Silverin [40] showed that great tits regain photosensitivity by mid-September in Southern Sweden, so we are confident that by December the birds from both populations were sensitive to long day lengths. Furthermore, expression of the clock genes Per2 and Cry1 showed the same pattern of oscillation during our sampling periods, suggesting that the two populations were entrained to similar photoperiods at the start of the experiment (Fig. 1). Instead, we suggest that differences in gene expression between the two populations relate to difference in their breeding ecology, in particular the differential importance of photoperiodic versus other environmental cues. In northern habitats, the time window when conditions are amenable for raising young begins later in the spring and is open for a shorter period of time [43]. Therefore, northern populations are predicted to rely almost exclusively on day length cues to time reproductive activity, and not make use of other cues such as temperature to the same extent as more southerly populations [3], [44], [45]. The finding that the German birds did not change gene expression in response to a single long day (except for FSHb, which occurred much later than in the Swedish population, see Fig. 2B) suggests that they either need to see multiple long days (i.e. require a long duration of photostimulation) or that they require multiple stimulatory cues at the same time, for example increases in temperature, food abundance, social stimuli. Alternatively (though not exclusively), German great tits might also be more sensitive to inhibitory cues (e.g., stress of captivity, lack of stimulatory visual cues, etc.).

### Conclusions

Our findings show that similar key genes are involved in PTM in a wild vertebrate as in domesticated species. Hence, the principle components of the PTM machinery exist across taxa, although the specific timing of gene expression appears to vary among species. Further studies on a diversity of wild species are required to determine whether these species differences are ecologically relevant. Our data cannot yet determine whether the population differences observed in this study are due to evolutionary or experiential differences between the two populations. However, they open up new avenues for future work examining ecological and evolutionary variation in the physiological organization of reproductive timing.

Finally, our data point to there being two distinct mechanisms regulating the photoperiodic response to a single long day. The first is the activation of the DIO2 system and rapid release of LH. The second is activation of the GnRH system which occurs 7 hours after the initial release of LH but provides prolonged stimulation of gonadotropin release. Focus on the search for an evolutionarily-conserved mechanism for photoperiodic timing has largely ignored activation (and deactivation) of the GnRH system. The combination of a lack of synchronization of DIO2 and GnRH activation along with the physical separation of the DIO2 and GnRH systems implies that photoperiodic up-regulation of GnRH might not involve the rapid activation of the DIO2 system in the geographically distinct mediobasal hypothalamus. This latter finding opens the door to a search for an additional mechanism for photoperiodic timing.

## Methods

### Ethics Statement

This study was performed under the approval of the state of Baden-Wuerttemberg, Germany, Animal Research Commission (protocol no. G-08/63).

### Animals

A total of 68 males (n = 33 Germany, n = 35 Sweden) were included in the study. Birds from both populations were captured in the fall (Oct/Nov) using passive mist netting and playback of male song to lure birds into mist nets. Males captured in Sweden were held in indoor, light-controlled aviaries 2–3 per cage until transport to Radolfzell, Germany by air on 1 Nov 08. In Germany, all birds were housed indoors in cages with artificial plants inside light-controlled environmental chambers 2–3 per cage. Birds were given food (egg food, sunflower seeds, mealworms) and water *ad libitum*. Birds were allowed to acclimate to the laboratory setting for five weeks.

### Experimental Design

During acclimation, birds were exposed to the same decreasing photoperiods that were similar to the site of capture. For German males, they received 10 hours 13 min of light at the end of October (lights on 0701 h: lights off 1714 h) decreasing to 8 hours 45 min of light by the end of November (lights on 0750 h: lights off 1635 h; a decrease of 1 hour 28 minutes). Swedish birds received 8 hours 30 min of light at the end of October (lights on 0800 h: lights off 1630 h) decreasing to 7 hours of light by the end of November (lights on 0830 h: lights off 1530 h; a decrease of one hour, see Fig. 4). Birds were fully photosensitive prior to photostimulation [40]. A blood sample only (no tissue was collected at this time point) was collected 24 hours before the start of the experiment at 6 hours after dawn (‘pre-experimental’), by puncturing the wing vein with a 26-gauge needle. Blood was collected into heparinized microcapillary tubes, plasma was separated with centrifugation and stored at −20C until assay. On 2 December lights were left on for the entire experiment and birds were sacrificed at 6 (German n = 5, Swedish n = 4) and at 10 (German n = 3, Swedish n = 3) hours after lights came on and these were pooled and called ‘pre-critical photoperiod’, also at 11.5 (German n = 6, Swedish n = 7; hereafter referred to as 11 h), 12.5 (German n = 5, Swedish n = 6; hereafter referred to as 12 h), 14 (German n = 5, Swedish n = 6), 18 (German n = 5, Swedish n = 6), and 22 (German n = 4, Swedish n = 4) hours after lights came on. Birds were sacrificed by decapitation after deep anesthesia with isoflurane (Phoenix Pharmaceuticals) and trunk blood was collected. Brain tissue, testis tissue and the remaining lower portion of the skull containing the anterior pituitary gland were quickly extracted and flash frozen on dry ice. We measured tarsus length with calipers to the nearest 0.10 mm and mass ±0.1 g. Fat was scored on a scale of 1 to 5 [46] in the furcular and abdominal region and a mean was calculated. Tissue was stored at −80C and shipped on dry ice to UC Berkeley.

**Table 1:** Primer sequences and anticipated size of the amplified products.

Gene	Forward Primer	Reverse Primer	bp	Genbank Accession #
PER2	TGTAGCTCTCTGGTCTCTGG	TACCTAGAGCTGCATGCTTC	104	JN797485
CRY1	AACAAGACTGGGGTGGTGAG	TGAGGAAGTTGATGGGGAAG	132	JN792410
DIO2	CAGGTCAAACTGGGAGGAGA	CACACTTGCCACCAACATTC	103	JN797486
DIO3	AGCTCAGCTGATGAGGGAAG	CTCGAAGTAGGCACCGTAGG	94	JN797487
GnRH	TGGGACCATTCCAGGAGATT	CTCTCCATGGCTTCCTTGAG	118	JN797488
FSHβ	CAGGATACTGCTTCACAAGG	TCTGACTGAAGGAGCAGTAG	240	JN797489
HPRT	GACCTGGACTTGTTCTGCAT	ATTTCACGTGCCAGTCTCTC	107	NM_204848
GAPDH	AGCAATGCTTCCTGCACTAC	CTGTCTTCTGTGTGGCTGTG	121	AF416452
PK-α	CCTATCCAAAATCGCTGTCC	AAGCGTGTTCCCTGATGTCT	120	NM_001012804
PK-τ	AAGAAGGACGTGGTGCTGAT	GTTTTCCTTGGTCTGGAACG	126	XM_002189768

### RT-PCR assay

#### Tissue processing

Brain tissue was cut on a cryostat beginning at approximately Plate A3.5 [47] when the tractus septomesencephalicus (TrSM) emerges for exactly 150 sections of 40 μm (3 mm in a rostral to caudal plane) until approximately Plate A0.5 (Fig. 5) from each brain. We sampled one 3mm circular tissue punch (Harris Uni-core, Electron Microscopy Sciences, cat#69036) on alternating right or left sides of each section. The edge of the circular punch was positioned just adjacent to the midline and ventral edge of the section. Punched tissue sections were mounted onto slides in pairs, so that each right and left side of the section had one punched and one adjacent intact section. These sections were used to verify, histologically, that we collected from the region of interest. This technique resulted in precisely equal total volume of tissue from each individual brain. Tissue punches were immediately added to 1ml TRIzol reagent (Invitrogen), homogenized and stored at −80C until extraction. Anterior pituitary tissue was extracted from the sella turcica using a dissecting microscope and immediately added to 1 ml TRIzol reagent, homogenized and stored at −80C until extraction.

#### Isolation of RNA and reverse transcription

Total RNA was isolated from TRIzol reagent in 200 μl chloroform. Total RNA was precipitated by adding 400 μl isopropanol followed by 80% ethanol washing, desiccation and reconstitution in 20 μl DEPC water. RNA quality and quantity were evaluated using a NanoDrop 2000 spectrophotometer. Following DNase treatment (DNA free, Ambion) to degrade any genomic DNA contamination, 12.5 μg of RNA from each sample was reverse transcribed to cDNA using M-MLV Reverse Transcriptase with oligo dT primers (Invitrogen). We diluted cDNA 1∶50 after testing serially diluted samples for each gene of interest to determine an optimal dilution.

#### qRT-PCR

Partial sequences for all of the genes of interest were first cloned from P. major cDNA (see Supplementary Materials S1). Primers were designed based on *P. major* sequences (using Primer3 software) for Per2, Cry1, DIO2, DIO3, GnRH, and FSHb (see Table 1 for primer sequences). Published *Gallus* sequences were used to design primers for control house-keeping genes: hypoxanthine phosphoribosyltransferase (HPRT), glyceraldehyde-3-phosphate dehydrogenase (GAPDH), protein kinase α, and protein kinase φ. Non-template controls were included for each primer pair to check for formation of primer-dimers. These samples always resulted in difference of at least 10 cycles of the Ct values compared to samples containing template. The quantitative real-time polymerase chain reaction (RT-PCR) was performed on samples balancing time points and populations across multiple plates using duplicate 25 μl reactions following manufacturer 's instructions for 2× iQ^TM^ SYBER Green Supermix (Bio-Rad Laboratories, Hercules, CA). Specificity of each primer pair was confirmed using a melt curve analysis. The raw fluorescent data were analyzed using the RT-PCR Miner program [48]. The PCR efficiency and fractional cycle threshold number were used for gene quantification. Expression values were calculated as 1/(1+E)∧Ct, where E is the average PCR efficiency and Ct is the cycle threshold. Four stable internal reference genes (HPRT, GAPDH, protein kinase α, and protein kinase φ) were used to normalize mRNA levels among samples. Two other reference genes were measured (18S and β-actin) and showed marked variation with photostimulation, so were not appropriate controls. We used GeNorm [49] to determine which reference genes were suitable and calculated a normalization factor for their expression. We then normalized the gene of interest expression by dividing expression values by the normalization factor for the controls. To calculate ‘fold change,’ we divided each normalized expression value by the minimum average expression value among the 12 means for each gene. This provides fold change above the minimum expression.

### Radioimmunoassay for LH

Plasma was assayed in 15 μl duplicate samples in a single assay using a micro-modification of the radioimmunoassay originally devised by Follett and colleagues [50]. Intra-assay variation was 3.2% and the lower detection limit was 0.07 ng/ml.

### Statistical analysis

We combined samples from 6 and 10 hours after dawn for each population because both represented ‘pre-threshold’ time points, expression levels were similar, and sample sizes at 10 hours (n = 3 per population) were especially low. These means are shown in graphs as ‘pre-threshold.’ We used student's t-tests to compare average morphology measures between populations. To test whether average mRNA expression values changed over time within populations or differed between populations at each time point, we used planned contrasts [51] using PASW Statistics 18.0. For gene expression data, we tested for differences between each time point and pre-threshold means, and between populations at each time point. For LH concentrations, we tested differences between log-transformed data at each time point and pre-bleed means, and between populations at each time point. Data are expressed as means ±1 standard error of the mean.

## Supporting Information

Supplementary Materials S1 **Methods used to partially clone genes of interest in** ***Parus major*** **.** These sequences were subsequently used to design primers for qPCR.
(DOCX)

## References

[1] Paul MJ, Zucker I, Schwartz WJ (2008). Tracking the seasons: the internal calendars of vertebrates.. Philos T R Soc B.

[2] Wingfield JC, Kenagy GJ (1991). Natural regulation of reproductive cycles..

[3] Wingfield JC, Jacobs JD, Tramontin AD, Perfito N, Meddle S, Wallen K, J S (2000). Toward an ecological basis of hormone-behavior interactions in reproduction of birds.. Reproduction in Context.

[4] Farner DS, Follett BK, Barrington EJW (1979). Reproductive periodicity in birds.. Hormones and Evolution.

[5] Murton RK, Westwood NJ (1977). Avian Breeding Cycles..

[6] Bronson FH, Heideman PD, Knobil E, Neill JD (1994). Seasonal regulation of reproduction in mammals.. The Physiology of Reproduction.

[7] Nakao N, Ono H, Yamamura T, Anraku T, Takagi T (2008). Thyrotrophin in the pars tuberalis triggers photoperiodic response.. Nature.

[8] Yoshimura T, Sharp PJ, Nelson RJ, Denlinger DL, Somers DE (2009). Genetic and molecular mechanisms of avian photoperiodism.. Photoperiodism: the biological calendar.

[9] Nicholls TJ, Follett BK (1974). Photoperiodic control of reproduction in Coturnix – quail temporal pattern of LH secretion.. J Comp Physiol.

[10] Revel FG, Saboureau M, Pevet P, Mikkelsen JD, Simonneaux V (2006). Melatonin regulates type 2 deiodinase gene expression in the Syrian hamster.. Endocrinology.

[11] Yoshimura T, Yasuo S, Watanabe M, Iigo M, Nakamura TJ (2007). Differential response of type 2 deiodinase gene expression to photoperiod between photoperiodic Fischer 344 and nonphotoperiodic Wistar rats.. Am J Physiol-Reg I.

[12] Hanon EA, Lincoln GA, Fustin JM, Dardente H, Masson-Pevet M (2008). Ancestral TSH mechanism signals summer in a photoperiodic mammal. Curr. Biol..

[13] Hazlerigg D, Loudon A (2008). New insights into ancient seasonal life timers. Curr. Biol..

[14] Yasuo S, Nakao N, Ohkura S, Iigo M, Hagiwara S (2006). Long-day suppressed expression of type 2 deiodinase gene in the mediobasal hypothalamus of the Saanen goat, a short-day breeder: implication for seasonal window of thyroid hormone action on reproductive neuroendocrine axis.. Endocrinology.

[15] Benoit J (1964). The role of the eye and of the hypothalamus in the photostimulation of gonads in the duck. Ann. N. Y. Acad. Sci..

[16] Yokoyama K, Oksche A, Darden TR, Farner DS (1978). The sites of encephalic photoreception in photoperiodic induction of the growth of the testes in the white-crowned sparrow, *Zonotrichia leucophrys gambelii*. Cell Tissue Res..

[17] Menaker M, Roberts R, Elliott J, Underwood H (1970). Extraretinal light perception in the sparrow, III: the eyes do not participate in photoperiodic photoreception.. P Natl Acad Sci USA.

[18] Foster RG, Grace MS, Provencio I, DeGrip WJ, Garcia-Fernandez JM (1994). Identification of vertebrate deep brain photoreceptors. Neurosci. Biobehav. Rev..

[19] Silver R, Witkovsky P, Horvath P, Alones V, Barnstable CJ (1988). Coexpression of opsin-like and VIP-like immunoreactivity in CSF-contacting neurons of the avian brain. Cell Tissue Res..

[20] Wada Y, Okano T, Adachi A, Ebihara S, Fukada Y (1998). Identification of rhodopsin in the pigeon deep brain. FEBS Lett..

[21] Yoshimura T, Nakane Y, Ikegami K, Ono H, Yamamoto N (2010). A mammalian neural tissue opsin (Opsin 5) is a deep brain photoreceptor in birds.. P Natl Acad Sci USA.

[22] Nakane Y, Ikegami K, Ono H, Yamamoto N, Yoshida S (2010). A mammalian neural tissue opsin (Opsin 5) is a deep brain photoreceptor in birds.. P Natl Acad Sci USA.

[23] Kang SW, Leclerc B, Kosonsiriluk S, Mauro LJ, Iwasawa A (2010). Melanopsin Expression in Dopamine-Melatonin Neurons of the Premammillary Nucleus of the Hypothalamus and Seasonal Reproduction in Birds.. Neuroscience.

[24] Halford S, Pires SS, Turton M, Zheng L, Gonzalez-Menendez I (2009). VA opsin-based photoreceptors in the hypothalamus of birds. Curr. Biol..

[25] Davies WIL, Turton M, Peirson SN, Follett BK, Halford S (2011). Vertebrate ancient opsin photopigment spectra and the avian photoperiodic response..

[26] Meddle SL, Follett BK (1997). Photoperiodically driven changes in Fos expression within the basal tuberal hypothalamus and median eminence of Japanese quail. J. Neurosci..

[27] Wada M (1979). Photoperiodic control of LH secretion in Japanese quail with special reference to the photoinducible phase. Gen. Comp. Endocrinol..

[28] Yamamura T, Yasuo S, Hirunagi K, Ebihara S, Yoshimura T (2006). T-3 implantation mimics photoperiodically reduced encasement of nerve terminals by glial processes in the median eminence of Japanese quail. Cell Tissue Res..

[29] Yamamura T, Hirunagi K, Ebihara S, Yoshimura T (2004). Seasonal morphological changes in the neuro-glial interaction between gonadotropin-releasing hormone nerve terminals and glial endfeet in Japanese quail.. Endocrinology.

[30] Bern HA, Nishoika RS, Mewaldt LR, Farner DS (1966). Photoperiodic and osmotic influences on the ultrastructure of the hypothalamic neurosecretory system of the white-crowned sparrow, *Zonotrichia leucophrys gambelii*. Z. Zellforsch..

[31] Farner DS, Wilson FE, Oksche A, Martini L, Ganong WF (1967). Neuroendocrine mechanisms in birds.. Neuroendocrinology.

[32] Follett BK, Mattocks PW, Farner DS (1974). Circadian Function in Photoperiodic Induction of Gonadotropin-Secretion in White-Crowned Sparrow, Zonotrichia-Leucophrys-Gambelii.. P Natl Acad Sci USA.

[33] Follett BK, Sharp PJ (1969). Circadian rhythmicity in photoperiodically induced gonadotrophin release and gonadal growth in the quail.. Nature.

[34] Hazlerigg DG, Wagner GC (2006). Seasonal photoperiodism in vertebrates: from coincidence to amplitude. Trends Endocrinol. Metab..

[35] Lewis RA (1975). Reproductive biology of the white-crowned sparrow. II. environmental control of reproductive and associated cycles.. Condor.

[36] Silverin B, Massa R, Stokkan KA (1993). Photoperiodic adaptation to breeding at different latitudes in great tits. Gen. Comp. Endocrinol..

[37] Ono H, Hoshino Y, Yasuo S, Watanabe M, Nakane Y (2008). Involvement of thyrotropin in photoperiodic signal transduction in mice.. Proc Natl Acad Sci U S A.

[38] Rubin CJ, Zody MC, Eriksson J, Meadows JRS, Sherwood E (2010). Whole-genome resequencing reveals loci under selection during chicken domestication.. Nature.

[39] Nicholls TJ, Follett BK, Robinson JE (1983). A Photoperiodic Response in Gonadectomized Japanese Quail Exposed to a Single Long Day. J. Endocrinol..

[40] Silverin B (1994). Photoperiodism in male great tits (Parus major). Ethol. Ecol. Evol..

[41] Watanabe T, Yamamura T, Watanabe M, Yasuo S, Nakao N (2007). Hypothalamic expression of thyroid hormone-activating and -inactivating enzyme genes in relation to photorefractoriness in birds and mammals.. Am J Physiol-Reg I.

[42] Yoshimura T, Yasuo S, Watanabe M, Iigo M, Yamamura T (2003). Light-induced hormone conversion of T-4 to T-3 regulates photoperiodic response of gonads in birds.. Nature.

[43] Baker JR (1938). The relation between latitude and breeding seasons in birds. Proc. Zool. Soc. Lond..

[44] Wingfield JC, Hahn TP, Doak D (1993). Integration of environmental factors regulating transitions of physiological state, morphology and behaviour..

[45] Wingfield JC, Hahn TP, Levin R, Honey P (1992). Environmental predictability and control of gonadal cycles in birds. J. Exper. Zool..

[46] Helms CW, Drury WH (1960). Winter and migratory weight and fat field studies on some North American buntings.. Bird Banding.

[47] Stokes TM, Leonard CM, Nottebohm F (1974). The telencephalon, diencephalon, and mesencephalon of the canary, *Serinus canaria*, in stereotaxic coordinates. J. Comp. Neurol..

[48] Zhao S, Fernald RD (2005). Comprehensive algorithm for quantitative real-time polymerase chain reaction. J. Comput. Biol..

[49] Vandesompele J, De Preter K, Pattyn F, Poppe B, Van Roy F (2002). Accurate normalization of real-time quantitative RT-PCR data by geometric averaging of multiple internal control genes.. Genome Biology.

[50] Follett BK, Farner DS, Mattocks PW (1975). Luteinizing hormone in the plasma of white-crowned sparrows, *Zonotrichia leucophrys gambelii*, during artificial photostimulation. Gen. Comp. Endocrinol..

[51] Gonzalez R (2009). Data analysis for experimental design..

