# Supplementary material for: Anticipating Spring: Wild Populations of Great Tits (Parus major) Differ in Expression of Key Genes for Photoperiodic Time Measurement
Source: PLoS One. 2012 Apr 23;7(4):e34997. doi: 10.1371/journal.pone.0034997 (PMC3334499; doi:10.1371/journal.pone.0034997)
Supplement: Supplementary Materials S1 — Methods used to partially clone genes of interest in Parus major . These sequences were subsequently used to design primers for qPCR. (DOCX) [file pone.0034997.s001.docx]

**Supplementary Materials S1**

*Partial cloning of P. major genes of interest*

We identified partial cDNA sequences encoding *Parus major* CRY1, PER2, DIO2, DIO3, GnRH (from hypothalamic tissue) and FSH-b (anterior pituitary gland tissue) following the methods of Ubuka et al. (2009). Total RNA was isolated by using a TRIZOL (Invitrogen, Carlsbad, CA) with chloroform extraction. Total RNA was reverse transcribed by using an oligo (deoxythymidine) 15 primer (Promega, Madison, WI) and reverse transcriptase (M-MLV Reverse Transcriptase; Invitrogen). *P. major* cDNA products were amplified with PCR by using various primers based on published sequences (see below). All PCR amplifications were performed in a reaction mixture containing Taq polymerase (TaKaRa Ex Taq; Takara Bio, Shiga, Japan). PCR products were subcloned into a pGEM-T Easy vector (Promega), and the DNA inserts of the positive clones were amplified by PCR with universal M13 primers. Amplified DNA was purified using (Exo-SAPit, Affymetrix), quantified, and sequenced at the University of California Berkeley DNA sequencing facility by using 3730xl DNA Analyzer (Applied Biosystems, Foster City, CA). Final gene of interest sequences compared to published sequences using BLAST and subsequently used to design *P. major* specific qRT-PCR primers.

We cloned a 1373 bp sequence of CRY1 using primers from *Sylvia borin* (AJ632120), forward primer 5’- GAGGATCTCGATGCCAATCT-3’ and reverse primer (5’-CCCATTAGACCACCATTTCC-3’). We used the published sequence for *Passer domesticus* (AY007259) to clone a 481 bp sequence of PER2 using forward primer 5’-CTCAGGATGTCAGCGTGTTC-3’ and reverse primer 5’-AAAGGTTGCCCTCCATACTG-3’. We used published *Gallus gallus* sequences (NM_204114 and NM_001122648) to clone DIO2 (448 bp) and DIO3 (549 bp). These included for DIO2, forward primer 5’-GGGTCTGTTAAGTGTGGA-3’ and reverse primer 5’-GCTCCTGGTATCTCCC-3’ and for DIO3, forward primer 5’-TTCTTCAAGTCGGCGCACGTGGGCT-3’ and reverse primer 5’-TTACACTTGGATGACCACCG-3’. We cloned a 322 bp sequence of GnRH using published sequences from Sturnus vulgaris (FJ514493), forward primer 5’-ACAACCTCTCTCAGGCA-3’ and reverse primer 5’-TCTGTGGCTCCATTT-3’. Finally, we used Gallus gallus sequence for FSH-b (NM_204257) to clone a 623 bp sequence using forward primer 5’- CAGGATACTGCTTCACAAGG-3’ and reverse primer 5’-GAGGAGAGATGGAAATGAGG-3’.
